# A Change Would Do You Good…or Would It? The Role of Emotion Variability in Female Adolescents’ Depressive Symptoms

**DOI:** 10.1007/s10802-025-01390-2

**Published:** 2025-10-21

**Authors:** Kirsten M. P. McKone, Kiera M. James, Cecile D. Ladouceur, Jennifer S. Silk

**Affiliations:** 1 Institute of Child Development, University of Minnesota, Minneapolis, MN USA; 2 Department of Psychology, University of Pittsburgh, Pittsburgh, PA USA; 3 Department of Psychiatry, University of Pittsburgh School of Medicine, Pittsburgh, PA USA

**Keywords:** Emotion variability, Emotion dynamics, Depression, Ecological momentary assessment

## Abstract

Depressive symptoms increase in adolescence, especially for female adolescents at risk for depression due to dispositional factors, such as temperament. Emotion dynamics, or change in emotional experience over time, may serve as a mutable mechanistic factor for depression. In a sample of 117 adolescents assigned female at birth ages 11–13 (*M*[SD] = 12.22[0.81], 68% white, 21% Black, 10% Hispanic/Latino, 9% biracial), oversampled for temperamental risk for the development of depression, this study examined emotion variability in association with depressive symptoms, both concurrently and longitudinally over an 18-month period. Further, this study extends the literature by examining associations between emotion variability and depressive symptoms by accounting for individuals’ typical levels of positive/negative emotion using two distinct methods: set-points or most frequent emotional state (i.e., mode adjustment) and average levels (i.e., mean adjustment). Results of mode-adjusted longitudinal growth curve models indicated that modal negative emotion, negative emotion variability, and positive emotion variability were all positively associated with adolescent females’ depressive symptoms at baseline but were not associated with change in depressive symptoms over time. By contrast, in mean-adjusted models, mean negative emotion was associated with baseline depressive symptoms, whereas variability in negative emotion was not. By contrast, only positive emotion variability was associated with depressive symptoms at baseline, whereas mean positive emotion was not. Neither was associated with change in depressive symptoms over time. Findings suggest that the putative difficulties with reactivity and regulation captured by emotion variability measures are related to adolescent females’ depressive symptoms – at least at non-clinical levels.

Rates of depression increase through adolescence (Avenevoli et al., 2015; Merikangas et al., 2010), and rates of adolescent depressive symptoms have increased substantially in the last decade (Keyes et al., 2019). Adolescents assigned female at birth and girls are at particular risk, as they experience increases in rates of depression at approximately twice the rate of youth assigned male at birth and boys (Hankin et al., 2015).^1^ Female adolescents at elevated risk for depression due to dispositional factors, such as shyness or fearfulness (Tortella-Feliu et al., 2010), are an especially important group to study, as the potential benefit of prevention and early intervention efforts is greater. Despite considerable scientific interest in preventing and ameliorating adolescent depression (Gotlib et al., 2020; Stockings et al., 2016), efforts have been largely unsuccessful at identifying mutable mechanistic factors that have significantly contributed to the identification of risk, improvement of prevention and intervention efforts, or substantially improved treatment response. Hence, identifying such mechanistic levers continues to be a primary focus of research (e.g., Goodman, 2020; Gotlib et al., 2020; Pagliaccio et al., 2020; Patil et al., 2018; Twenge, 2020).

## Emotion Variability as a Mechanism in the Development of Depression

Researchers have long looked to changes in emotion as possible mechanisms associated with depression onset. Early research focused on mean-level emotion alterations in depression, specifically higher levels of negative emotion (NE) and lower levels of positive emotion (PE; e.g., Watson et al., 1988). More recently, scholars have investigated *change* in emotion over time and examined *dynamic* emotion as a potential mechanism of depression onset (Hamaker & Wichers, 2017; Houben et al., 2015; Kuppens & Verduyn, 2017; Wichers et al., 2015), including overall change in emotion (e.g., variability), differences in the stability of emotion (e.g., instability), and increased *resistance* to change in emotion (e.g., inertia).

Altered emotion dynamics may be a mechanism in the development of depression in female adolescents – especially when normative developmental changes in emotion in adolescence are considered in conjunction with the increase in incidence of depression during adolescence. Adolescents exhibit more NE, less PE, and more mood variability on average than children/adults (Griffith et al., 2021; Larson et al., 2002; Larson & Lampman-Petraitis, 1989; Maciejewski et al., 2015; Reitsema et al., 2022), with one study showing that mood variability decreases from age 13 through age 18 (Maciejewski et al., 2015). Developmental changes in broad emotional experience appear more pronounced in female adolescents and girls: Compared to boys, girls experience steeper increases in NE and steeper/more prolonged decreases in PE throughout adolescence (Abitante et al., 2022; Griffith et al., 2021). These systematic alterations in both static and dynamic measures of emotion in adolescence may indicate that normative emotion development has gone awry in female adolescents who go on to develop psychopathology. Alterations in emotion dynamics in adolescence may be especially relevant for depression, considering normative adolescent changes in emotion mirror the alterations in emotion observed in clinical depression. Indeed, a meta-analysis by Reitsema and colleagues (2022) found adolescents with psychopathology had more intense anxiety, greater sadness variability, and more variable and less intense PE compared to adolescents without psychopathology, alterations that appeared to be more severe deviations from the normative developmental changes in emotion observed in the study.

The alterations in dynamic emotion experience observed during adolescence may be driven, in part, by increased neural sensitivity to social information (Blakemore & Mills, 2014; Crone & Dahl, 2012). Peers become increasingly influential during adolescence and sensitivity to social feedback is associated with increased variability in both PE and NE (Somerville & Casey, 2010). Additionally, adolescents become increasingly independent at managing their own emotions and are exposed to a wider array of contexts than in childhood. These developmental and contextual factors provide strong support for considering altered emotion dynamics as a possible mechanism for the development of depression in female adolescents.

### Associations between Emotion Variability and Depression in Adolescence

One emotion dynamics construct that has been leveraged in research on the development of depression in adolescence is *emotion variability*, defined as change over time in one’s emotion level across a range of contexts. Higher levels of emotion variability are theorized to reflect broad failures of reactivity and regulation, which may consolidate into clinically altered emotional function, such as is observed in depression (Houben et al., 2016). Laboratory studies of dynamic emotion in adolescence suggest that altered emotion dynamics (at the timescale of seconds to minutes) are implicated in the development and maintenance of depressive symptoms in adolescence (Koval et al., 2012; Kuppens et al., 2010, 2012). Studies of emotion variability have also been conducted using ecologically sensitive methods, (e.g., ecological momentary assessment [EMA], daily diary) to capture adolescent emotional experience in daily life (e.g., Neumann et al., 2011; Silk et al., 2003). Although laboratory methods facilitate the examination of adolescent emotion dynamics in a more rigorously controlled manner, ecologically valid methods like EMA present a valuable opportunity to advance understanding of dynamic emotion processes as they unfold in adolescents’ real-world contexts (i.e. in the presence of peers, parents, and realistic emotion-inducing situations).

Several studies have found that higher levels of NE variability are associated with elevated depressive symptoms (e.g., low/irritable mood, changes in appetite/sleep, excessive guilt) and diagnosis (Maciejewski et al., 2014, 2019; Neumann et al., 2011; Silk et al., 2003, 2011). Beyond concurrent associations, there is evidence that NE variability may play a mechanistic role in depressive symptom maintenance in adolescence (Neumann et al., 2011; Silk et al., 2011). However, several studies have shown that NE variability is no longer a significant predictor of depressive symptoms above and beyond mean levels of NE, likely due to high levels of confounding between the mean and variability (Ringwald & Wright, 2022). Fewer studies have examined associations between depressive symptoms and PE variability, with some studies demonstrating associations between internalizing symptoms and PE variability (Morgan et al., 2017; van Roekel et al., 2016) and other studies not (Forbes et al., 2012). However, research suggests that PE alterations may be relatively unique to depression amongst internalizing disorders, compared to alterations in NE, which are more broadly shared across internalizing psychopathology (Forbes et al., 2004; although see Sequeira et al., 2022), pointing to a potential role for PE variability in the development of anxiety symptoms.

Despite these initial indications of a positive relationship between emotion variability and depressive symptoms, variation in study methodology underlines the need for further investigation. Studies have differed in timescale (hours vs. days; Morgan et al., 2017; Silk et al., 2003, 2011; van Roekel et al., 2016; Maciejewski et al., 2014, 2019; Neumann et al., 2011), and whether they examine NE and PE variability separately (e.g., Silk et al., 2011; Morgan et al., 2017) or in conjunction (e.g., “mood” variability; e.g., Neumann et al., 2011). Finally, studies have differed in the extent to which they adjusted for measures of central tendency, with some studies including no adjustment (e.g., Silk et al., 2003) and some adjusting for average levels of PE/NE (e.g., Maciejewski et al., 2014). Some scholars argue that emotion dynamics measures add limited predictive benefit beyond measures mean-level emotion and advocate for the mean as the strongest predictor of symptoms/disorder (Dejonckheere et al., 2019). However, others have more recently argued that the high degree of dependence between the mean and variabiltiy measures is a confound, and advocate for the use of the mode as a more clincially relevant “set-point” of affect (Ringwald & Wright, 2022). However, to date, no studies have investigated both methods (i.e., adjustment for mean vs. mode) in the same study in adolescence.

## The Current Study

The current study seeks to clarify associations between emotion variability and depressive symptoms in adolescence in a sample of female adolescents enriched for dispositional risk for the development of depression. Further, we test these associations considering two different forms of emotional central tendency: modal levels of emotion, or individuals’ most frequent emotional “set-point” (Ringwald & Wright, 2022), and mean levels of emotion, or individuals’ average levels of NE/PE, as has been typically used in the literature.

Consistent with prior literature (Maciejewski et al., 2014, 2019; Neumann et al., 2011; Silk et al., 2003, 2011), we hypothesized that higher NE variability in daily life (i.e., assessed via EMA) would be associated with higher levels of depressive symptoms in female adolescents at baseline (i.e., positive association with intercept), and greater increases in depressive symptoms across the 18-month follow-up (i.e., positive association with slope), above and beyond effects of modal emotion, which we hypothesized would follow the same pattern (i.e., higher modal NE associated with more depressive symptom intercept and slope). In models testing NE variability with mean NE, we predicted higher mean NE would be associated with more baseline depressive symptoms (i.e., positive association with intercept) and greater increases in symptoms over time (i.e., positive association with slope). Turning to PE variability, although there is relatively less research examining the association, due to the relatively unique role of altered PE in depression (Forbes et al., 2004), we hypothesized that PE variability would be positively associated with depressive symptoms concurrently (i.e., intercept) and across 18-month follow-up (i.e., slope), beyond the effects of modal levels of PE, which we predicted would be associated with fewer concurrent depressive symptoms and less increase in symptoms over time (i.e., negative association with intercept and slope). By contrast, in models testing PE variability with mean PE, we predicted only higher mean PE would be associated with fewer baseline depressive symptoms (i.e., negative association with intercept) and less increase in symptoms over time (i.e., negative association with slope).

## Method

### Participants

Participants were drawn from the first wave of the GIRLS Brain Study which was approved by the Institutional Review Board at the University of Pittsburgh. Advertisements and community postings were used to recruit early adolescents assigned female at birth ages 11–13 for a longitudinal study of neural and social factors in the development of depression and anxiety in adolescents assigned female at birth. Five hundred fifty-two families completed an initial phone/web screening. Two hundred and thirty-five adolescent females met criteria and 197 participants completed the initial screening visit. As the study was designed to examine risk for the development of anxiety and depression, exclusion criteria included current diagnosis or history of DSM-5 anxiety disorder (except specific phobia, due to the narrower scope and decreased functional impairment) and/or major depressive disorder, to ensure participants did not already have a history of anxiety or depression and facilitate the interpretation of results in the context of development of these disorders. The study also excluded for severe psychopathology (diagnoses of bipolar disorder, schizophrenia, or acute suicidality) due to the extreme alterations in emotional experience often characteristic of these disorders. Participants with diagnoses of the hyperactive-impulsive and combined types of attention-deficit/hyperactivity disorder were also excluded due to concerns about movement during an fMRI scanning protocol not pertinent to the present analysis. Additional exclusion criteria included: a diagnosis of autism spectrum disorder; IQ < 70 (assessed with the two-scale Wechsler Abbreviated Scale of Intelligence; Wechsler, 1999); current or past neurological or serious medical condition; fMRI contraindications; uncorrected impaired vision; past head injury or neurological anomalies; and current use of medication that impacts the central nervous system. All enrolled participants provided informed consent (from parents/guardians) and informed assent (from adolescents). The overarching study was designed to have adequate power (β = 0.80, α < 0.05) to detect medium-sized effects. A separate power analysis for the current study was not conducted.

The final sample for the overarching study comprised 129 female adolescents. Female sex assigned at birth was parent-reported. Approximately two-thirds of participants were at elevated risk for social anxiety and depression based on elevated dispositional shyness/fearfulness (i.e., scored 0.75 *SD* or higher than the sample mean on the Fear and/or Shyness subscales of the Early Adolescent Temperament Questionnaire-Revised; EATQ-R; Ellis & Rothbart, 2001). As noted below, 12 participants were excluded from analyses based on missing the EMA or not meeting minimum response thresholds during the EMA period and some participants had missing data on depression outcomes, resulting in a final analysis sample of *n* = 117 early adolescents.

## Procedure

The study procedures were approved by the institutional review board at the University of Pittsburgh. The parent study occurred in three-waves with brief questionnaire follow-ups at 6-month intervals. At wave 1, of primary pertinence to this study, participants engaged in three lab visits. At the first visit, adolescents and a caregiver (typically biological mothers) completed online questionnaires and a clinical interview, as study exclusion criteria included the presence of several clinical diagnoses, as noted above. At the second visit (approximately one month after their first lab visit), adolescents completed several individual tasks and a series of dyadic interaction tasks with their participating caregiver. After the second laboratory visit, adolescents completed a 16-day EMA protocol starting the Saturday following their second laboratory visit. (The third laboratory visit was an fMRI scan. As those procedures are not relevant to the current report, they will not be discussed further here.) Adolescents and caregivers also completed brief questionnaires assessing anxiety and depressive symptoms at 6-month intervals. This study leverages reports from baseline, 6-month, 12-month, and 18-month follow-up assessments.

### Ecological Momentary Assessment

At the end of the second lab visit, adolescents were provided with a study smartphone and instructed on completing EMA on a range of emotional, social, and behavioral topics, most of which are not relevant to the current report. Participants were prompted three times on weekdays (early morning prior to school, after school, and evening) and four times on weekend days (morning, early afternoon, late afternoon, evening) for 16 consecutive days (six weekend days, ten weekdays) for a maximum of 54 observations. Prompts were randomly delivered within three-hour blocks in the timeframes described above, with the exception of the morning prompt which was delivered in a morning window set by the participant based on their waketime. Surveys took seven minutes to complete on average; participants received reminders every 15 min for up to an hour, after which time the survey expired.

## Measures

### Emotion Variability

At the beginning of each EMA survey, participants were asked to report their current mood on eight emotion indicators: happy, sad, joyful, worried, stressed, interested, mad, excited. Each emotion was rated on a 0–100 sliding scale where *0* = *Not at all* and *100* = *Extremely*, with the slider initially anchored at 0. To create PE and NE variability composites, the highest of the four indicators of each broad emotion measure (PE = happy, joyful, interested, excited; NE = sad, worried, stressed, mad) was selected within each observation to create an overall PE/NE value for that observation.^2^ Reliability was adequate for both NE (*α*_within_ = 0.67, *α*_between_ = 0.94; *ω*_within_ = 0.68, *ω*_between_ = 0.95) and PE (*α*_within_ = 0.80, *α*_between_ = 0.94; *ω*_within_ = 0.80, *ω*_between_ = 0.94). Within-person *SD*s of momentary PE/NE (i.e., each observation) were calculated as a measure of PE/NE variability. Further, mean and modal PE/NE, respectively, were included to represent individuals’ average/set-points of PE/NE (Ringwald & Wright, 2022).

### Adolescent Depressive Symptoms

At the baseline visit and 6-, 12-, and 18-month follow-up, female adolescents completed the Mood and Feelings Questionnaire (Messer et al., 1995), a 33-item measure designed to assess symptoms of depression, via an online Qualtrics survey. The MFQ was not designed to have a strict clinical cutoff; however, psychometric studies have found that a score of 29 effectively discriminated clinical cases of depression. Reliabilities ranged from ⍺ = 0.89–0.95.

### Covariates

Age and socioeconomic status were evaluated as covariates in light of established associations with depression (e.g., Abitante et al., 2022; Griffith et al., 2021; Hankin et al., 2015; Maciejewski et al., 2014; Peverill et al., 2021). Age at the baseline visit was calculated in years by subtracting the participant’s birth date from their visit date. Socioeconomic status was approximated by including parent-reported annual gross income in dollars on a 0–10 scale, where 0 = 0–10,000 and 10 = 100,000+. Both age and socioeconomic status were group-mean centered to facilitate interpretation.

### Analytic Approach

Analyses were conducted in R version 4.2.1 (R Core Team, 2022) using the nlme package (Pinheiro et al., 2025) and Mplus Version 7.31 (Muthen & Muthen, 2023).

### Model Specification

All models were tested using maximum likelihood estimation to address missing data. Depressive symptom sum scores were used as indicators of a longitudinal growth curve model to capture depressive symptom trajectories, with the intercept fixed at baseline. Iterative model testing was conducted to ensure models were correctly specified. Model improvement was tested (where possible) with a −2 log likelihood test. A means-only model with a random intercept of participant was run first, to examine the decomposition of the variance at the between- and within-person levels, followed by the addition of fixed and random effects of linear time. We evaluated effect sizes by use of pseudo-R^2^, following recommendations by Cohen (2013) for effect sizes in multiple regression of small (*R*^2^ = 0.02), medium (*R*^2^ = 0.15), or large (*R*^2^ = 0.35).

Finally, to test the contributions of each predictor of interest (e.g., PE/NE variability) on depressive symptoms, a series of models were fit. The longitudinal growth model of depressive symptoms served as the baseline model. Once the fit of the longitudinal growth model was established, covariates were added to the model. Covariates were tested sequentially in the baseline model; if the covariate was not a significant predictor of depressive symptoms, the covariate was not retained. (Although see below regarding missingness related to socioeconomic status.) For models testing PE/NE variability, modal (or mean) PE/NE was added first, to test incremental improvement in model fit with the addition of emotion variability beyond the adjustment for central tendency.

### Missing Data

One participant did not complete the depression measure at any timepoint and was therefore excluded. Additionally, seven participants were missing the measure assessing self-reported depressive symptoms at the 6-month assessment, nine at the 12-month assessment, and 11 at the 18-month assessment. Twelve participants were missing or excluded from the EMA portion of the current study: One participant withdrew from the study, one was unable to participate in EMA, two had technical problems, and eight did not meet compliance criteria (completed fewer than 25% of prompts, gave primarily nonsense answers). Included participants completed 78% of prompts on average. The final analysis sample comprised *n* = 117 participants.

We evaluated patterns of missingness on depressive symptom assessments according to the recommendations outlined by Hayes and colleagues (Hayes et al., 2024). We examined associations between possible auxiliary variables (all predictors and covariates, plus shyness, fearfulness, and baseline parent depressive symptoms) and missingness in depressive symptom reports at any wave of assessment. We first conducted *t-*tests of all possible auxiliary variables, which indicated significant associations between socioeconomic status and modal NE and depressive symptom missingness, such that participants with lower socioeconomic status and lower modal NE were more likely to be missing on one of the follow-up depression assessments. When all possible auxiliary variables were included in a logistic regression, no variables were significant predictors of missingness (all *p*s > 0.05). We also ran logistic regression models testing all linear interaction and quadratic terms to test for non-linear sources of missingness; no predictors were significant (all *p*s > 0.05). Finally, we ran a model including only socioeconomic status and modal NE; in that model, socioeconomic status was marginally significantly predictive of missingness (*p* =.058) and modal negative emotion was no longer significant (*p* =.994). As a result, we assumed data were missing at random and included socioeconomic status as a covariate in all models.

## Results

Descriptive statistics are included in Table 1. Means, standard deviations, and bivariate correlations between study variables are included in Table 2. Depressive symptoms ranged from 0 to 30 at baseline, suggesting that few, if any, participants had clinically significant depressive symptoms at the outset of the study (consistent with study procedures targeted at recruiting a non-clinical sample with elevated risk for the development of depression). At later waves, depressive symptoms ranged into the likely clinical level of 29 or greater (Range_6m_ = 0–39; Range_12m_ = 0–32; Range_18m_ = 0–40), and 1, 2, 3, and 2 participants were above the clinically discriminant level of the MFQ at baseline, 6 m, 12 m, and 18 m, respectively.

**Table 1:** Descriptive statistics by sample

	Analysis Sample				Full Sample			
Variable	*N/n*	*M/%*	*SD*	Range	*N/n*	*M/%*	*SD*	Range
Age (Years)	117	12.22	0.81	11.05–13.98	128	12.27	0.80	11.05–13.98
Race	116				128			
White, not Hispanic	80	69%	-	-	87	68%	-	-
Black, not Hispanic	22	19%	-	-	26	20%	-	-
Biracial	10	9%	-	-	11	9%	-	-
Asian	2	1%	-	-	2	2%	-	-
Native American	1	1%	-	-	1	1%	-	-
Other	1	1%	-	-	1	1%	-	-
Ethnicity	113				125			
Hispanic	10	9%			10	8%		
Not Hispanic	103	91%			115	92%		
Socioeconomic Status	114	7.12	3.16	0–10	126	7.09	3.20	0–10
Fear subscale (EATQ-R)	115	2.28	0.73	1–3.83	127	2.32	0.73	1–3.83
Shyness subscale (EATQ-R)	115	2.74	0.95	1–5	127	2.73	0.98	1–5
Pubertal Development (PDS)	115	3.32	0.79	1–5	128	3.36	0.77	1–5

Percentages may not add up to 100% due to rounding. EATQ-R = Early Adolescent Temperament Questionnaire-Revised (Ellis & Rothbart, 2001). PDS = Pubertal Development Scale (Petersen, 1998). Socioeconomic status was measured on a 0–10 scale of annual gross income in dollars where 0 = 0–10,000 and 10 = 100,000+. The participant who endorsed a racial identity of “Other” self-identified as white, Black/African American, and American Indian/Native American

**Table 2:** Means, standard deviations, and correlations of study variables

Variable	M	1	2	3	4	5	6	7	8	9	10	11
1. Age	12.25(0.82)											
2. SES	6.99(3.29)	0.14										
		[−0.05, 0.32]										
3. NE mode	17.52(17.64)	0.17	0.10									
		[−0.02, 0.35]	[−0.10, 0.29]									
4. PE mode	74.57(26.42)	− 0.02	0.02	− 0.14								
		[−0.21, 0.18]	[−0.17, 0.21]	[−0.33, 0.05]								
5. NE mean	18.59(16.24)	0.15	− 0.01	0.79**	− 0.03							
		[−0.04, 0.33]	[−0.20, 0.19]	[0.71, 0.86]	[−0.22, 0.16]							
6. PE mean	67.91(19.19)	0.02	0.09	− 0.17	0.88**	− 0.10						
		[−0.17, 0.22]	[−0.11, 0.28]	[−0.35, 0.03]	[0.82, 0.91]	[−0.29, 0.09]						
7. NE variability	19.55(8.84)	0.01	− 0.22*	0.19*	0.20*	0.56**	0.13					
		[−0.18, 0.20]	[−0.40, − 0.03]	[0.00, 0.37]	[0.01, 0.38]	[0.42, 0.68]	[−0.07, 0.31]					
8. PE variability	18.53(7.88)	− 0.10	− 0.14	0.11	− 0.30**	0.19	− 0.46**	0.25**				
		[−0.29, 0.09]	[−0.32, 0.06]	[−0.09, 0.29]	[−0.47, − 0.12]	[−0.00, 0.37]	[−0.60, − 0.30]	[0.06, 0.42]				
9. Dep. sx T0	9.41(7.18)	− 0.07	− 0.18	0.27**	0.02	0.33**	0.04	0.33**	0.25**			
		[−0.25, 0.11]	[−0.35, 0.00]	[0.08, 0.44]	[−0.18, 0.21]	[0.15, 0.49]	[−0.15, 0.23]	[0.15, 0.49]	[0.06, 0.42]			
10. Dep. sx 6 m	7.73(7.73)	0.01	0.05	0.42**	− 0.08	0.38**	− 0.13	0.18	0.24*	0.51**		
		[−0.18, 0.20]	[−0.14, 0.24]	[0.25, 0.57]	[−0.27, 0.12]	[0.21, 0.54]	[−0.32, 0.07]	[−0.02, 0.36]	[0.05, 0.41]	[0.35, 0.63]		
11. Dep. sx 12 m	8.98(8.24)	0.05	0.05	0.28**	− 0.13	0.36**	− 0.15	0.23*	0.26**	0.51**	0.64**	
		[−0.14, 0.24]	[−0.14, 0.24]	[0.09, 0.45]	[−0.32, 0.07]	[0.17, 0.52]	[−0.33, 0.05]	[0.03, 0.40]	[0.07, 0.43]	[0.35, 0.64]	[0.52, 0.74]	
12. Dep. sx 18 m	9.22(8.59)	0.06	0.11	0.37**	− 0.07	0.38**	− 0.15	0.09	0.17	0.32**	0.57**	0.63**
		[−0.14, 0.25]	[−0.09, 0.30]	[0.19, 0.53]	[−0.27, 0.13]	[0.20, 0.54]	[−0.34, 0.05]	[−0.11, 0.28]	[−0.03, 0.35]	[0.13, 0.48]	[0.42, 0.69]	[0.50, 0.73]

*M* and *SD* are used to represent mean and standard deviation, respectively. Values in square brackets indicate the 95% confidence interval for each correlation. The confidence interval is a plausible range of population correlations that could have caused the sample correlation. * indicates *p* <.05. ** indicates *p* <.01. *SES* socioeconomic status, *NE* negative emotion, *PE* positive emotion, *Dep. sx* depressive symptoms

We then evaluated bivariate correlations, following Cohen’s (2013) recommendation for correlations of small (*R*^2^ = 0.10), medium (*R*^2^ = 0.30), or large (*R*^2^ = 0.50) effect size. At the bivariate level, the mean and mode of NE and PE, respectively, were highly positively correlated within-valence at a very large effect size. Modal NE was positively correlated with NE variability at a small effect size, whereas mean NE and NE variability were positively correlated at a medium to large effect size. Modal PE was negatively correlated with PE variability at a small-to-medium effect size, whereas mean PE and PE variability were negatively correlated at a medium effect size. NE and PE variability were positively correlated at a small effect size, indicating that participants who reported more variability in NE also reported more PE variability. PE mode was positively associated with NE variability at a small effect size, indicating that participants who reported higher “set-points” of PE also reported more variability in NE. Depressive symptoms were moderately positively correlated across assessments. Higher NE means and modes were associated with more depressive symptoms at each wave at small to medium effect sizes. More PE variability was also associated with more depressive symptoms at the first three assessments.

### Longitudinal Growth Curve Models

Iterative model testing was performed to identify the best-fitting, most parsimonious model. Model fit statistics are in Table 3. First, a means-only model of depressive symptoms was assessed, including a random intercept for participant. The intraclass correlation coefficient (ICC) indicated just over half of the variance was at the between-person level (ICC = 0.53) and a little less than half was at the within-person level. Second, a fixed effect of time was added, and was not a significant predictor of depressive symptoms, indicating there was not a significant linear increase or decrease on average across the 18-month span (*B* = 0.02, *p* =.637). Third, a random linear effect of time was added. A −2 log likelihood test indicated the model significantly improved; however, the intraclass correlation coefficient indicated there was relatively little variance accounted for by the random slope of time (ICC = 0.002).

**Table 3:** Model fit statistics

Model	*N*	Number of Parameters	AIC	BIC	−2LL
Measurement Model					
1. Random Intercept	128	2	3,233	3,245	3,226
2. Fixed Growth	128	3	3,234	3,251	3,226
3. Random Growth	128	6	3,221	3,246	3,208
4. Covariate Model	126	7	3,170	3,204	3,154
Mode Adjustment					
1a. NE mode	114	8	2,913	2,953	2,892
1b. NE variability	114	9	2,910	2,955	2,888
2a. PE mode	114	8	2,934	2,975	2,914
2b. PE variability	114	9	2,925	2,970	2,902
Mean Adjustment					
3a. NE mean	114	8	2,910	2,951	2,890
3b. NE variability	114	9	2,911	2,956	2,890
4a. PE mean	114	8	2.932	2,973	2,912
4b. PE variability	114	9	2,925	2,970	2,902

*NE* negative emotion, *PE* positive emotion

### Covariates

Covariates were tested individually, both as direct effects and in interaction with time (i.e., testing differences in slope dependent on age). Age was not significantly associated depressive symptoms either independently (*p* =.512) or in interaction with time (*p* =.185). Socioeconomic status was not significantly associated with depressive symptoms intercept (*p* =.116) but significantly interacted with depressive symptom slope (*B* = 0.03, *p* =.018), indicating that participants with lower socioeconomic status had more negative slopes.

### Emotion Variability

**Fig. 1:**
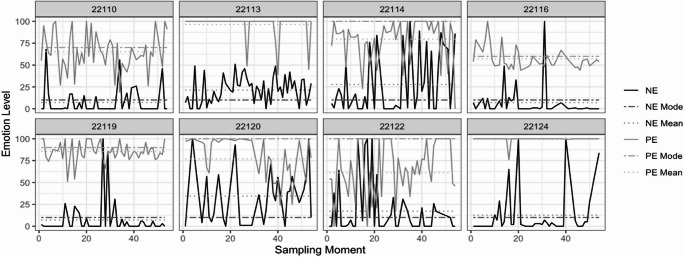
NE and PE Mean, Mode and Variability for a Subset of Participants*. Note*: Each panel depicts the time series for a single participant. NE = negative emotion, PE = positive emotion

A representative sample of participant means, modes and change in emotion over the course of the 16-day EMA period is depicted in Fig. 1. First, NE mode was entered into the model as a direct effect and in interaction with time. Modal NE significantly predicted depressive symptoms at a medium effect size, but did not interact with depressive symptom slope (see Table 4). Second, NE variability was added as a direct effect and in interaction with time. NE variability significantly positively predicted depressive symptoms at a small effect size, but did not interact with depressive symptom slope, indicating that higher levels of change in NE over the EMA period were associated with higher baseline levels of depressive symptoms but not with change in depressive symptoms over time.

**Table 4:** Model results for NE variability with adjustment for modal and average NE

	Depressive Symptoms											
	Model 1			Model 2			Model 3			Model 4		
*Predictors*	*B*	*95% CI*	*p*	*B*	*95% CI*	*p*	*B*	*95% CI*	*p*	*B*	*95% CI*	*p*
Intercept	**9.66**	**6.59–12.73**	**<** **0.001**	**5.83**	**1.50–10.15**	**0.009**	**8.42**	**5.18–11.66**	**<** **0.001**	**7.10**	**2.64–11.56**	**0.002**
Time	**−0.30**	**−0.51 – −0.09**	**0.005**	−0.18	−0.48–0.13	0.256	**−0.32**	**−0.54 – −0.10**	**0.005**	−0.18	−0.48–0.13	0.253
Socioeconomic Status	**−0.43**	**−0.81 – −0.04**	**0.030**	−0.32	−0.71–0.06	0.103	−0.33	−0.71–0.05	0.091	−0.30	−0.69–0.09	0.137
Time x Socioeconomic Status	**0.04**	**0.01–0.06**	**0.004**	**0.04**	**0.01–0.06**	**0.007**	**0.04**	**0.01–0.07**	**0.002**	**0.04**	**0.01–0.06**	**0.007**
NE Mode	**0.13**	**0.07–0.20**	**<** **0.001**	**0.13**	**0.07–0.18**	**<** **0.001**						
Time x NE Mode	0.00	−0.00–0.01	0.577									
NE Mean							**0.16**	**0.08–0.23**	**<** **0.001**	**0.16**	**0.08–0.25**	**<** **0.001**
Time x NE Mean							0.00	−0.00–0.01	0.446			
NE Variability				**0.17**	**0.03–0.31**	**0.022**				0.05	−0.12–0.22	0.563
Time x NE Variability				−0.00	−0.01–0.00	0.357				−0.00	−0.01–0.00	0.359
**Random Effects**												
s^2^	24.22			24.23			24.24			24.23		
t_00_	24.72 Individual			22.66 Individual			24.28 Individual			24.10 Individual		
t_11_	0.05 Individual: time			0.05 Individual: time			0.05 Individual: time			0.05 Individual: time		
r_01_	−0.05 Individual			0.00 Individual			−0.08 Individual			−0.06 Individual		
ICC	0.55			0.54			0.54			0.54		
N	114 Individual			114 Individual			114 Individual			114 Individual		
Observations	443			443			443			443		
Marginal R^2^/Conditional R^2^	0.132/0.611			0.143/0.608			0.145/0.610			0.137/0.606		

*NE* negative emotion. Values in bold are significant at *p* <.05

Next, we entered PE mode into the baseline longitudinal growth curve model as a direct effect and in interaction with depressive symptom slope. PE mode was not significantly associated with depressive symptoms at baseline or in interaction with depressive symptom change over time (Table 5). We then added PE variability and its interaction with depressive symptom slope. PE variability was significantly positively associated with depressive symptoms at baseline at a small to medium effect size, but was not associated with changes in depressive symptom slope (i.e., no significant interaction with time), indicating that higher levels of change in positive emotion during the EMA was associated with higher initial levels of depressive symptoms.

**Table 5:** Model results for PE variability with adjustment for modal and average PE

	Depressive Symptoms											
	Model 1			Model 2			Model 3			Model 4		
*Predictors*	*B*	*95% CI*	*p*	*B*	*95% CI*	*p*	*B*	*95% CI*	*p*	*B*	*95% CI*	*p*
Intercept	**12.63**	**7.96–17.29**	**< 0.001**	5.66	−0.29–11.60	0.064	12.23	**6.93–17.53**	**< 0.001**	4.10	−3.02–11.22	0.262
Time	−0.26	−0.56–0.04	0.086	−0.24	−0.53–0.05	0.114	−0.11	−0.45–0.23	0.532	−0.24	−0.53–0.06	0.114
Socioeconomic Status	−0.33	−0.74–0.08	0.120	−0.24	−0.64–0.15	0.231	−0.32	−0.73–0.09	0.126	−0.25	−0.64–0.15	0.219
Time x Socioeconomic Status	**0.04**	**0.01–0.06**	**0.003**	**0.04**	**0.01–0.06**	**0.004**	**0.04**	**0.02–0.07**	**0.002**	**0.04**	**0.01–0.06**	**0.004**
PE Mode	−0.02	−0.07–0.03	0.471	0.00	−0.04–0.05	0.918						
Time x PE Mode	−0.00	−0.00–0.00	0.890									
PE Mean							−0.01	−0.08–0.05	0.684	0.02	−0.05–0.09	0.540
Time x PE Mean							−0.00	−0.01–0.00	0.222			
PE Variability				**0.26**	**0.10–0.42**	**0.002**				**0.28**	**0.11–0.45**	**0.001**
Time x PE Variability				−0.00	−0.01–0.01	0.685				−0.00	−0.01–0.01	0.683
**Random Effects**												
σ^2^	24.17			24.21			24.15			24.21		
τ_00_	30.87 _Individual_			26.64 _Individual_			31.04 _Individual_			26.27 _Individual_		
τ_11_	0.05 _Individual: time_			0.05 _Individual: time_			0.05 _Individual: time_			0.05 _Individual: time_		
ρ_01_	0.01 _Individual_			0.04 _Individual_			−0.01 _Individual_			0.06 _Individual_		
ICC	0.61			0.58			0.60			0.58		
N	114 _Individual_			114 _Individual_			114 _Individual_			114 _Individual_		
Observations	443			443			443			443		
Marginal R^2^/Conditional R^2^	0.016/0.612			0.072/0.611			0.021/0.611			0.074/0.612		

*PE* positive emotion. Values in bold are significant at *p* <.05

### Variability Analyses with Adjustment for Mean Emotion

Models for emotional variability were also run using mean affect as an adjustment for central tendency. All models fit adequately (Table 3). Socioeconomic status remained a significant predictor of depressive symptom slope in both models (Tables 4 and 5).

Mean NE significantly predicted depressive symptoms at baseline at a medium effect size, but did not interact with change in depressive symptoms over time (Table 4). When mean NE was included as the adjustment for central tendency, NE variability was not significantly associated with depressive symptoms at baseline or over time. Mean PE was not significantly associated with depressive symptoms at baseline or over time (Table 5). By contrast, PE variability was significantly positively associated with depressive symptoms at baseline at a small to medium effect size, indicating that participants who reported higher levels of change in PE during EMA had higher baseline depressive symptoms.

## Discussion

This study examined emotion variability as a predictor of depressive symptoms in a sample of early adolescent females, examining associations using two methods of adjusting for typical emotion level: average (mean) levels of PE/NE, and individual set-points (mode) of PE/NE. Consistent with hypotheses, more changeability in emotion throughout the course of daily life was associated with higher initial levels of depressive symptoms in early adolescent females. After accounting for the fact that female adolescents with higher “set points” of NE (i.e., modal NE) reported more initial depressive symptoms, more change in female adolescents’ negative emotions during their daily lives was associated with higher initial levels of depressive symptoms. Additionally, participants who experienced wider and/or more frequent swings in PE during daily life also reported higher initial depressive symptoms. These findings are consistent with the literature on emotion dynamics, which indicates that more emotion variability is broadly associated with psychopathology, including depression (Houben et al., 2015; Larson & Csikszentmihalyi, 1980; Maciejewski et al., 2015, 2019; Neumann et al., 2011). An important caveat is that the range of depressive symptoms was constrained in this sample, partly by design, as the study aimed to examine predictors of the *development* of depression, and partly due to relatively limited increase in depression over time, which was unexpected. As a result, findings are broadly limited in generalizability to samples of subclinical depressive symptoms.

As adjustment for the mode is a novel approach, we separately adjusted for the effects of mean-level emotion. Adjusting for the mean facilitates comparison with other studies, and provides an alternative conceptualization of variability and “steady state” emotion. Results suggested that, consistent with previous studies, mean NE is a stronger predictor of depressive symptoms than NE variability, when both are included in the model. By contrast, more PE variability was associated with more baseline depressive symptoms across both methods of adjustment. These results provide additional justification for the thoughtful selection of measures of central tendency to “adjust” for typical emotion levels in examinations of affective variability, as differing implications emerged dependent on operationalization.

The associations between depressive symptoms and emotion variability found here align with broad alterations in emotion in depression, that is, higher mean NE and lower mean PE (Watson et al., 1988). Whereas set-points of NE were associated with initial levels of depressive symptoms, only PE variability, not set-points of PE, was associated with initial levels of symptoms. It may be that the association between emotion variability and depressive symptoms at baseline indicates that increased emotion variability in early adolescence is an early signal of risk for the onset of depression later in adolescence; longitudinal studies are needed to identify the direction of effect between emotion variability and depressive symptoms.

The emotion variability observed here may reflect the reorganization of emotion during a pivotal developmental period, at least for female adolescents (Crone & Dahl, 2012). The female adolescents in this sample typically reported very low levels of NE and very high levels of PE during their everyday lives, suggesting the positive association between NE variability and depressive symptoms reflects *larger increases or spikes* in NE from moment to moment, whereas the positive association between PE variability and depressive symptoms reflects *larger decreases or drops* in PE from a relatively high set point. Continued variability in NE from a low set-point may coalesce into a higher NE set point, whereas continued variability in PE from a high set-point may coalesce into a lower PE set-point over time, consistent with dynamic systems theories of perturbations and attractors (Wichers et al., 2015). The physical and hormonal changes of puberty may function as such a perturbation, spurring reorganization of adolescent females’ emotional experience through hormonally mediated alterations in mood (e.g., Moore et al., 2012; Silk et al., 2009). Unfortunately, most of the female adolescents in this sample had relatively advanced pubertal status (i.e., post-menarcheal), precluding investigation of this possibility here. Longitudinal examinations across pubertal development will be necessary to test these hypotheses. Further, longitudinal research examining emotion variability and change in depressive symptoms in females in a cross-lagged panel framework (or similar) would allow for the teasing out of the “chicken and egg” of these two processes, which are likely bidirectional at least to some extent.

There are several possible explanations for the PE variability findings. Experiencing more change in PE, particularly in the form of drops from a high set-point, may be experienced as brief periods of anhedonia. It is plausible to imagine female adolescents experiencing lower PE in everyday situations eventually electing not to engage in those activities, potentially leading to withdrawal and further suppressed PE (Silk et al., 2012). Alternately, developmental changes and/or individual differences in emotional reactivity to social feedback (Somerville & Casey, 2010), reward responsivity (Forbes & Dahl, 2012), or the interaction of the two (i.e., social sensitivity and reward responsivity) may be associated with the suppression of reward systems in response to repeated frustrated social rewards (Davey et al., 2008; Silk et al., 2012), and subsequent increased PE variability.

Notably, these explanations do not extend to NE variability. Alternative explanations are that variability is distressing in its own right, consistent with theories of a drive for homeostasis (Plutchik, 1982) and contrast avoidance theory (Newman & Llera, 2011), or consequences from affective deviance (Kastendieck et al., 2021; Szczurek et al., 2012), mechanistic explanations that would potentially account for associations between both PE and NE variability and depressive symptoms. Although these are examples of possible underlying mechanisms, research is needed to elucidate the underlying and likely interconnected biological, environmental, and psychological factors related to such oscillations and their role in depression in female adolescents. It is also possible that NE and PE variability are not fully distinct processes, but rather interrelated to some degree, as suggested by associations between depressive symptoms and “mood variability” across both positive and negative emotions. Further psychometric and methodological research is needed to illuminate the respective contributions of valence-specific and valence-agnostic measures of emotion variability. Similarly, alternative models of dynamic emotion that integrate changes in PE and NE, such as socioaffective flexibility and state-space grid approaches (Hollenstein, 2015; McKone & Silk, 2022) may provide additional insights into the role of dynamic emotion processes in the development of adolescent depression.

### Clinical Implications & Future Directions

In addition to adjusting for mean-level emotion, the current study adjusted for an emotional “set point” in conjunction with variability (Ringwald & Wright, 2022), which may aid in understanding variability in emotion in a more ecologically valid and clinically relevant manner. Emotional deviations from one’s set-point may better capture the full “swing” of change in emotion experienced from moment to moment in someone’s life, which may have implications for the perceived severity of change and falicitate the development of more targeted intervention. Further, leveraging modal emotion reinforces the importance of the contextual changes that prompt changes in emotion. Collecting and examining contextual correlates of an individual’s modal emotional state and deviation from that modal state is an important future direction for emotion variability research (Houben et al., 2015; McKone & Silk, 2022), in order to disentangle context-specific and individual difference-mediated emotion change. For example, perhaps female adolescents report higher modal NE but more stability with family and lower modal NE but more variability with peers; such insights would promote more sensitive questions regarding the relation between emotion variability and psychopathology that would more directly inform clinical intervention, such as “What contextual changes are associated with larger changes in emotion and for whom?”

Similarly, collecting data on female adolescents’ attributions in concert with their emotion ratings would facilitate research into cognitive processes that may be especially potent influences and potential prime targets for intervention. Identifying contextual or attributional factors associated with larger deviations from set-points may facilitate the design of strategic interventions; for example, if many female adolescents experience more variability at school, in the evening hours, or when they are with particular people, such contexts may be effective targets for delivering personalized, just-in-time interventions, such as emotion regulation strategies or grounding exercises, via mobile phone.

Socioeconomic status may be one broader context that is important to consider with regard to the development of depressive symptoms. Notably, female adolescents with higher socioeconomic status exhibited more positive slopes of depressive symptoms over time. The sample studied here was of relatively high socioeconomic status, so these findings may be more relevant in the context of middle- to upper-income adolescents, and the findings likely would not generalize to populations with higher rates of participants in poverty, where higher socioeconomic status would likely have a protective effect (Peverill et al., 2021).

### Limitations

There are several limitations to note. First, these findings are likely inflated by shared informant bias, as adolescents self-reported both depressive symptoms and emotion. Repeating these analyses with clinician-rated symptoms, ideally based on both adolescent and parent report, is an important next step. Further, findings should be considered in the context of possible heterogeneity in depression and/or nonlinear associations (Santee & Starr, 2021); it is plausible that *very low* levels of emotion variability may be indicative of one phenotype of depression (Bylsma,  2021; Koval et al., 2012; Kuppens et al., 2012), and that maintaining moderate levels of emotion variability is most adaptive, indicating appropriate response to contextual changes. Research testing these questions is required. This study was also likely underpowered for the prediction of change in depressive symptoms over time, as symptom levels were low at baseline, and remained relatively stable throughout the course of the study. In other words, there was limited variability in growth in depressive symptoms over time, which was unexpected given the study design (i.e., risk for development of depression). Finally, although the sample collected here is broadly representative of female adolescents and families in the midsize Midwestern city in which it was collected, it is approximately two-thirds white, so findings are not generalizable to populations with higher proportions of racial and ethnic minority groups or to adolescent males.

## Conclusion

Findings suggest that experiencing more change in emotions in daily life may be a prodromal process in the development of depression in female adolescents, particularly in the context of variability in PE, which was robust across two methods of adjusting for individuals’ emotional tendency. However, more research leveraging developmentally informed methods is needed to tease out the direction of effects.

## Data Availability

Data, materials, and code are available upon request to the corresponding author.

## Abbreviations

NE: negative emotion
PE: positive emotion
EMA: ecological momentary assessment

## Footnotes

1 When discussing evidence of sex and gender differences in depression in the literature, we have attempted to be clear in terms of the precise construct of sex or gender used. However, not all studies were explicit about their method of measurement or operationalization of sex/gender; in such cases, we have used the term that best reflects the level of inference, or both when study measurement was unclear.

2 The maximum negative/positive emotion at each timepoint was used rather than averaging across all four indicators as a result of visual inspection of the data. Participants seldom reported non-zero values across multiple emotions (compared to reporting a non-zero value on just one emotion per valence), and if they did so, they often selected very similar values (e.g., 25 on all four emotions). Using the maximum value at each timepoint allowed the retention of the strongest emotion reported, which also allowed emotion ratings to remain on the original 0-100 scale, easing interpretation. We evaluated the correlation between the mean and the max emotion at each observation and found that the measures are very highly correlated (*r*_*PE*_*=*0.88; *r*_*NE*_ *=*0.93), suggesting that the methods produce nearly identical results.
